# Synthesis and characterization of thermally stable aromatic polyamides and poly(1,3,4-oxadiazole-amide)s nanoparticles containing pendant substituted bezamides

**DOI:** 10.1186/1752-153X-7-13

**Published:** 2013-01-23

**Authors:** Hammed HAM Hassan, Amel F Elhusseiny, Yasmeen MA Elkony, El-Sayed ME Mansour

**Affiliations:** 1 Chemistry Department, Faculty of Science, Alexandria University, P.O. Box 426-Ibrahimia, Alexandria 21321, Egypt

**Keywords:** Nanoparticles, Synthesis, Aramides, Fluorescence, SEM, Thermal analysis, Oxadiazole ring

## Abstract

**Background:**

The introduction of pendent bulky groups along the polymer backbone results in a less ordered polymer matrix and increases the solubility characteristics without affecting thermal properties. The inclusion of chromogenic chemical moieties in the chains can give rise to the luminescent converter material which permits the preparation of materials with potential applications. Aromatic polymers containing heterocyclic rings in the main chain are known for their high thermal resistance, good hydrolytic stability, low dielectric and tough mechanical properties. There is currently much research directed towards the discovery of new blue light-emitting polymers, with characteristics of high efficiency and high reliability. Herein, we describe the preparation of aromatic polyamides and poly (1,3,4-oxadiazole-amide)s nanoparticles with pendant structures comprised of *m*- and *p*-acetoxybenzamide groups, where the acetoxybenzamide groups act as signaling units due to their fluorescent and chromogenic characteristics.

**Results:**

Aromatic polyamides and poly(1,3,4-oxadiazole-amide)s nanoparticles with pendant structures comprised of *m*- and *p*-acetoxybenzamide groups were successfully prepared and characterized using different analytical methods. Most polyamides were obtained as well-separated spherical nanoparticles while aramide containing pyridine produced aggregated particles attributed to the molecular self assembly via H-bond directed organization of molecular precursors. The thermal behavior of all polymers exhibited two major thermal decompositions due to the subsequent breakage of the acetoxy group in the lateral chain and cleavage of the main amide bonds. Photoluminescence studies revealed that the blue emissions for the polyamide derived from benzidine were blue-shifted (shifted to a lower wavelength) compared to that of polyamides containing flexible linkages.

**Conclusions:**

We report the synthesis of aromatic polyamides and poly(1,3,4-oxadiazole-amide)s nanoparticles with pendant structures comprised of *m*- and *p*-acetoxybenzamide groups. The thermal behavior of all polymers exhibited two major decompositions due to breakage of the acetoxy group in the lateral chain and cleavage of the main amide bonds. Structure- photoluminescence correlation demonstrated an interesting connection between structural modification and optical properties. The blue emissions for the polyamide derived from benzidine, attributed to the highly conjugation system, was blue shifted with the introduction of flexible linkages. The prepared polymers dissolved in warm polar aprotic solvents. Further investigations to obtain films with reasonably good mechanical properties for different applications are in progress.

## Background

Chemical functionalization of the structure of high-performance materials, that is, aramids, expands their applicability to new and cutting-edge fields. Chemical modification of aromatic polyamides is rarely performed. Few successful examples have been published in the literature due to their insolubility and the presence of amide linkages that can undergo side reactions. Several approaches to soluble polyamides including the incorporation of flexible linkages or bulky substitutes have been developed [1]. The main concept behind all these approaches is the reduction of the packing force and the increasing of the free volume of the polymers. Methods used to improve the solubility and lower glass transition temperature of the polymers are the introduction of flexible groups, large pendent groups or polar substituents. The introduction of pendent bulky groups along the polymer backbone results in a less ordered polymer matrix increasing the solubility characteristics without affecting thermal and mechanical properties to any great extent. The design and preparation of organic functional materials can be carried out by two methods [2,3]: by synthesis of monomers containing the desired functionality or by modification of the chemical structure of a parent polymer. The latter has advantages related to the economics of the process and it opens a way for total or partial modification of the parent structure with the desired functionality while still retaining the properties of the parental compound to a degree including the characteristics of the functional groups. Thus, the inclusion of chromogenic and fluorescent chemical moieties in the lateral aromatic polyamide chains [1], can give rise to the luminescent converter (LUCO) materials, which in combination with a primary pumping source, that is, blue light-emitting diodes (LEDs), permits the preparation of LEDs with tremendous potential in lighting and backlighting applications [4-8]. In addition, if the chromogenic and fluorescent moieties include a substructure capable of interacting selectively with analytes, the variations in the chromogenic or fluorescent behavior of the material permit the preparation of chemical sensors that could be used to detect analytes using the different spectral characteristics of light emitted by hybrid LUCO/LED devices or by fine tuning the visual perception of their emitted light (color). In parallel, if the functionalization gives rise to colored host substructures, the interaction with guest analytes may also lead to color changes, which could give rise to “naked eye” colorimetric sensing materials [9,10].

Heterocyclic rings have been widely incorporated into the chains of the polyamides. Generally, the heterocyclic units in the main chain and/or in the pendant structure impart excellent thermal stability with improved solubility which justifies the research efforts directed toward them [11-14]. Aromatic polymers containing 1,3,4-oxadiazole rings in the main chain are well known for their high thermal resistance in oxidative atmosphere, good hydrolytic stability, low dielectric constant and tough mechanical properties [15-17]. There is currently much research directed towards the discovery of new blue light-emitting polymers, with characteristics of high efficiency and high reliability. For such a purpose poly(1,3,4-oxadioazole)s are of great interest due to electron withdrawing character of the 1,3,4-oxadiazole rings that can facilitate the injection and transport of electrons. Several different reaction pathways have been developed to prepare poly(1,3,4-oxadiazole)s [18]. The most popular synthesis involves the preparation of a precursor polyhydrazides by the reaction of a diacyl chloride or derivative with hydrazine or a dihydrazide compound. The precursor polyhydrazids are cyclized to the polyoxadiazoles by heating under vacuum or heating in a dehydrating solvent such as sulfuric acid, polyphosphoric acid, or phosphoryl chloride. A different synthetic procedure produces polyoxadiazoles in one step by the solution polymerization of a dicarboxylic acid or the corresponding nitrile, amide, or ester with hydrazine or its salt in polyphosphoric or sulfuric acid or a phosphorus pentoxide/methanesulfonic acid mixture. In addition, aromatic polyether synthesis through aromatic nucleophilic displacement reaction has been used for the preparation of aryl ether-containing poly(1,3,4 oxadiazole)s or 1,3,4-oxadiazole-containing polyethers.

We previously succeeded to prepare aromatic polyamides nanoparticles with remarkable electrical and biomedical properties [19-23]. Herein, we describe the preparation of novel aromatic polyamides and poly (1,3,4-oxadiazole-amide)s nanoparticles with pendant structures comprised of *m*- and *p*-acetoxybenzamide groups [11], where the acetoxybenzamide groups act as signaling units due to their fluorescent and chromogenic characteristics. These model compounds were also used to study the influence of the acetoxy group orientation on the thermal stability and photoluminescence behavior of the polymers.

## Experimental

### Materials

5-Aminoisophthalic acid, *m*-hydroxybenzoic acid, *p*-hydroxylbenzoic acid and the commercial diamines namely; *m*-phenylenediamine, *p*-phenylenediamine, 2,6-diaminopyridine, benzidine, 4,4^′^- oxydianiline, 4,4^′^-dianilinesulfone, 4,4^′^-methylenedianiline and the solvents *N,N*-dimethyl acetamide (DMAc), *N,N*-dimethylformamide (DMF), dimethylsulfoxide (DMSO) (Alfa), methyl alcohol (Aldrich), thionylchloride (Alfa), acetic anhydride (Aldrich), hydrazine hydrate (Aldrich), acetyl chloride (Aldrich) were used as purchased without purification.

### Measurements

Melting points were determined with an electro-thermal melting point apparatus and are not corrected. Infrared spectra (IR, KBr pellets; 3 mm thickness) were recorded on a Perkin-Elmer Infrared Spectrophotometer (FTIR 1650). All spectra were recorded within the wave number range of 600–4000 cm^-1^ at 25°C. Absorption spectra were measured with a UV 500 UV—Vis spectrometer at room temperature (rt) in DMSO with a polymer concentration of 2 mg /10 mL. Inherent viscosities (*η*_inh_) were measured at a concentration of 0.5 g/dL in DMSO at 30°C by using an Ubbelohde viscometer. Differential thermogravimetric (DTG) analyses were carried out in the temperature range from 25°C to 700°C in a steam of nitrogen atmosphere by a Shimadzu DTG 60H thermal analyzer. The experimental conditions were: platinum crucible, nitrogen atmosphere with a 30 ml/min flow rate and a heating rate 20°C/min. Differential scanning calorimetry (DSC-TGA) analyses were carried out using SDTQ600-V20.5-Build-15 at the Institute of Graduate Studies and Research, Alexandria University. Elemental analyses were performed at the Microanalytical Unit, Cairo University. The morphologies of polymer nanoparticles were observed by Scanning Electron Microscope (SEM) (JEOL-JSM5300), at the E-Microscope Unit; Faculty of Science, Alexandria University. The samples were sonicated in de-ionized water for 5 min and deposited onto carbon coated copper mesh and allowed to air-dry before the examination.

### Synthesis of 5-(4-acetoxybenzoylamino)isophthaloyl chloride 10

To a 50 ml round bottom flask were added *p*-hydroxyl benzoic acid **1** (10 g, 72.5 mmol) and acetic anhydride (15 ml). The mixture was refluxed for 2 h and the product was crystallized from cold water. The solid was collected by filtration, washed with large amount of water and crystallized from chloroform. *p*-Acetoxybenzoic acid **3** was obtained as a white crystal in 84% yield (10.9 g); m.p. 190°C.

*p*-Acetoxybenzoic acid **3** (5 g, 27.6 mmol) and thionyl chloride (25 ml) were refluxed for 1 h (till complete dissolution of the starting material). The excess of thionyl chloride was removed under vacuum. The desired *p*-acetoxybenzoyl chloride **5** was obtained as a white solid in 83% yield (5.5 g).

5- Aminoisophthalic acid **7** (5 g, 72.5 mmol) dissolved in DMA (30 ml) was well stirred and then *p*-acetoxybenzoyl chloride **5** (5.5 g, 27.6 mmol) was added. The mixture was stirred for 20 h at rt and then it was poured into water and filtered. The required 5-(4-acetoxybenzoylamino) isophthalic acid **8** was obtained in 95% yield (9 g); m.p. 296°C. IR: 3286, 3077, 2567, 1758, 1720, 1694, 1656, 1602, 1539, 1505, 1439, 1412, 1372, 1334, 1283, 1203, 1168, 1107, 1070, 1016, 951, 914, 883, 854, 758, 694, 666, 612, 539, 502, 464.

Finely powdered diacid **8** (6 g, 17.5 mmol) was mixed with thionyl chloride (25 ml). The mixture was refluxed for 1 h and then distilled off. The desired 5-(4-acetoxybenzoylamino)isophthaloyl chloride **10** was obtained in quantitative yield (7.2 g); m.p. 126°C and it was used as such in the following steps.

### Synthesis of 5-(3-acetoxybenzoylamino)isophthaloyl chloride 11

*m*-Acetoxybenzoic acid **4** was obtained starting from *m*-hydroxylbenzoic acid **2** as a white crystal in 78% yield; m.p. 130°C, following the above described method for preparation of compound **3**. *m*-Acetoxybenzoyl chloride **6** was obtained in 83% yield starting from compound **4** following the above described method for preparation of compound **5**.

5-(3-Acetoxybenzoylamino)isophthalic acid **9** was obtained in 89% yield (8.4 g) as a white solid starting from coupling reaction of 5-aminoisophthalic acid **7** and *m*-acetoxybenzoyl chloride **6** following the above described method for preparation of compound 8. IR (υ, cm^-1^): 3299, 2987, 2830, 2640, 2569, 1761, 1690, 1656, 1588, 1538, 1487, 1446, 1408, 1373, 1334, 1277, 1214, 1135, 1105, 1078, 1016, 1001, 957, 930, 914, 836, 809, 759, 693, 679, 614, 533, 510, 470, 440.

Finely powdered diacid **9** (6 g, 17.5 mmol) was mixed with thionyl chloride (25 ml) and the mixture was refluxed for 1 h. The mixture was distilled off and the obtained solid of 5-(3-acetoxybenzoylamino)isophthaloyl chloride **11** was used as such without further purification. IR: 3901, 3852, 3837, 3292, 3078, 2922, 2850, 1952, 1762, 1653, 1586, 1540, 1482, 1450, 1370, 1335, 1294, 1199, 1143, 1016, 999, 948, 903, 877, 833, 805, 783, 684, 607, 528, 494, 470.

### Polymer synthesis by low temperature solution polycondensation (General method)

In a dry round flask, the appropriate diamine (10.0 mmol) and 5-(4-acetoxybenzoylamino)isophthaloyl chloride **11** (10.0 mmol) in 15 ml of DMA was strongly stirred at 0°C for 30 min. then at rt for 10 h. The solution was poured into cooled water and the produced polymer was collected by filtration and washed subsequently with water, ethanol and water again and then dried in a vacuum oven at 60°C.

#### Reaction of 5-(4-acetoxybenzoylamino)isophthaloyl chloride 10 with p-phenylenediamine: Preparation of Polymer 12

Yield: 62.0%, m.p. > 300°C, *η*_inh_ = 0.575 dL/g. IR (υ, cm^-1^): 3289 (N-H_str_), 3078, 2580, 1715 (CO ester), 1657 (CO amide), 1601 (aromatic C-H_str_, 1548, 1506, 1430, 1371, 1332, 1281, 1203, 1167, 1107, 1016, 951, 914, 882, 855, 758, 693, 668, 612, 518. Elemental analysis calculated for **12** (C_23_H_19_N_3_O_6_)_n_: C, 63.74; H, 4.38; N, 9.69; Found: C, 63.29; H, 4.02; N, 9.40.

#### Reaction of 5-(3-acetoxybenzoylamino)isophthaloyl chloride 11 with p-phenylenediamine: Preparation of Polymer 13

Yield: 71.4%, m.p. > 300°C, *η*_inh_ = 0.575 dL/g. IR (υ, cm^-1^): 3897, 3863, 3848, 3832, 3814, 3794, 3743, 3729, 3705, 3684, 3664, 3641, 3290, 3072, 2921, 2355, 1902, 1762, 1737, 1659, 1599, 1552, 1514, 1445, 1403, 1370, 1313, 1218, 1110, 1015, 978, 900, 832, 748, 680, 599. Elemental analysis calculated for **13**, (C_23_H_19_N_3_O_6_)_n_: C, 63.74; H, 4.38; N, 9.69; Found: C, 63.39; H, 4.81; N, 9.28.

#### Reaction of 5-(4-acetoxybenzoylamino)isophthaloyl chloride 10 with m-phenylenediamine: Preparation of Polymer 14

Yield: 62.0%, m.p. > 300°C, *η*_inh_ = 0.672 dL/g. IR (υ, cm^-1^): 3303 (N-H_str_), 1748, 1659 (C = O amide), 1603 (aromatic C-H_str_), 1542, 1488, 1445, 1369, 1336, 1302, 1201, 1166, 1109, 1015, 915, 884, 855, 784, 757, 687, 614, 543. Elemental analysis calculated for **14** (C_23_H_19_N_3_O_6_)_n_: C, 63.74; H, 4.38; N, 9.69. Found: C, 63.81; H, 4.77; N, 9.75.

#### Reaction of 5-(3-acetoxybenzoylamino)isophthaloyl chloride 11 with m-phenylenediamine: Preparation of Polymer 15

Yield: 60%, m.p. > 300°C, *η*_inh_ = 0.445 dL/g. IR (υ, cm^-1^): 3686, 3299, 3078, 2923, 2358, 1760, 1738, 1661, 1604, 1546, 1485, 1443, 1369, 1333, 1296, 1217, 1015, 886, 830, 784, 747, 685, 601, 533. Elemental analysis calculated for **15** (C_23_H_19_N_3_O_6_)_n_: C, 63.74; H, 4.38; N, 9.69; Found: C, 64.11; H, 4.56; N, 9.83.

#### Reaction of 5-(4-acetoxybenzoylamino)isophthaloyl chloride 10 with 2,6-diaminopyridine: Preparation of Polymer 16

Yield: 50%, m.p. > 300°C, *η*_inh_ = 0.765 dL/g. IR (υ, cm^-1^): 3351, 1658, 1604, 1552, 1505, 1447, 1370, 1333, 1281, 1201, 1165, 1015, 912, 853, 800, 759, 670, 611. Elemental analysis calculated for **16** (C_22_H_18_N_4_O_6_)_n_: C, 60.82; H, 4.14; N, 12.90; Found: C, 60.42; H, 3.88; N, 12.61.

#### Reaction of 5-(3-acetoxybenzoylamino)isophthaloyl chloride 11 with 2,6-diaminopyridine: Preparation of Polymer 17

Yield: 50%, m.p. > 300°C, *η*_inh_ = 0.575 dL/g. IR (υ, cm^-1^): 3986, 3849, 3831, 3813, 3792, 3702, 3302, 3070, 2921, 2357, 1762, 1735, 1659, 1595, 1530, 1445, 1410, 1370, 1321, 1214, 1112, 1017, 904, 810, 750, 682, 604, 531. Elemental analysis calculated for **17** (C_22_H_18_N_4_O_6_)_n_ : C, 60.82; H, 4.14; N, 12.90; Found: C, 60.54; H, 4.53; N, 13.27.

#### Reaction of 5-(4-acetoxybenzoylamino)isophthaloyl chloride 10 with benzidine: Preparation of Polymer 18

Yield: 95%, m.p. > 300°C, *η*_inh_ = 0.575 dL/g. IR (υ, cm^-1^): IR: 3434, 1756, 1618, 1503, 1403, 1319, 1244, 1195, 1166, 1060, 1018, 912, 851, 821, 742, 674, 595, 517, 479. Elemental analysis calculated for **18** (C_29_H_23_N_3_O_6_)_n_: 68.36; H, 4.51; N, 8.25. Found: C, 67.98; H, 4.91; N, 7.89.

#### Reaction of 5-(3-acetoxybenzoylamino)isophthaloyl chloride 11 with benzidine: Preparation of Polymer 19

Yield: 83%, m.p. > 300°C. IR (υ, cm^-1^): 3639, 3301, 3071, 2924, 2357, 1903, 1762, 1735, 1661, 1592, 1501, 1445, 1415, 1369, 1322, 1293, 1213, 1112, 1005, 900, 819, 747, 680, 599, 517. Elemental analysis for **19** (C_29_H_23_N_3_O_6_)_n_: C, 68.36; H, 4.51; N, 8.25; Found: C, 68.72; H, 4.89; N, 7.99.

#### Reaction of 5-(4-acetoxybenzoylamino)isophthaloyl chloride 10 with 4,4'-oxydianiline: Preparation of Polymer 20

Yield: 51%, m.p. > 300°C, *η*_inh_ = 0.472 dL/g. IR (υ, cm^-1^): 3303, 3070, 1748, 1657, 1600, 1539, 1499, 1445, 1408, 1370, 1310, 1285, 1230, 1166, 1106, 1014, 960, 914, 876, 832, 757, 702, 613, 515. Elemental analysis calculated for **20** (C_29_H_23_N_3_O_7_)_n_: C, 66.28; H, 4.38; N, 8.00; Found: C, 66.72; H, 4.66; N, 8.33.

#### Reaction of 5-(3-acetoxybenzoylamino)isophthaloyl chloride 11 with 4,4'-oxydianiline: Preparation of Polymer 21

Yield: 88%, m.p. > 300°C, *η*_inh_ = 0.448 dL/g. IR (u, cm^-1^): 3303, 3070, 2926, 2854, 2359, 2337, 2075, 1883, 1763, 1740, 1661, 1599, 1535, 1499, 1444, 1409, 1369, 1297, 1230, 1106, 1043, 1014, 945, 876, 831, 747, 703, 682, 598, 513. Elemental analysis calculated for **21** (C_29_H_23_N_3_O_7_)_n_: C, 66.28; H, 4.38; N, 8.00; Found: C, 65.99; H, 4.13; N, 8.38.

#### Reaction of 5-(3-acetoxybenzoylamino)isophthaloyl chloride 11 with 4,4'-methylenedianiline: Preparation of Polymer 23

Yield: 71%, m.p. > 300°C, *η*_inh_ = 0.419 dL/g. IR (υ, cm^-1^): 3986, 3849, 3831, 3813, 3792, 3702, 3302, 3070, 2921, 2357, 1762, 1735, 1659, 1595, 1530, 1445, 1410, 1370, 1321, 1214, 1112, 1017, 904, 810, 750, 682, 604, 531, 511. Elemental analysis calculated for **23** (C_30_H_25_N_3_O_6_)_n_: C, 68.83; H, 4.78; N, 8.03; Found: C, 68.46; H, 5.02; N, 7.44.

#### Reaction of 5-(4-acetoxybenzoylamino)isophthaloyl chloride 10 with 4,4'-diaminodiphenylsulphone: Preparation of polymer 24

Yield: 51%, m.p. > 300°C, *η*_inh_ = 0.575 dL/g. IR (υ, cm^-1^): 3367, 3103, 1919, 1749, 1667, 1592, 1527, 1445, 1400, 1369, 1317, 1252, 1202, 1166, 1148, 1105, 1072, 1015, 956, 914, 835, 755, 718, 691, 629, 574, 554. Elemental analysis calculated for **24** (C_29_H_23_N_3_O_8_S)_n_: C, 60.73; H, 4.01; N, 7.32; Found: C, 61.09; H, 4.32; N, 7.71.

#### Reaction of 5-(3-acetoxybenzoylamino)isophthaloyl chloride 11 with 4,4'-diaminodiphenylsulphone: Preparation of polymer 25

Yield: 53%, m.p. > 300°C, *η*_inh_ = 0.445 dL/g. IR (υ, cm^-1^): 3473, 3361, 3102, 2925, 2359, 2338, 1904, 1763, 1738, 1668, 1591, 1529, 1445, 1400, 1369, 1318, 1218, 1148, 1105, 1072, 1014, 943, 902, 835, 749, 719, 688, 628, 591, 554. Elemental analysis calculated for **25** (C_29_H_23_N_3_O_8_S)_n_: C, 60.73; H, 4.01; N, 7.32; Found: C, 60.46; H, 3.87; N, 7.64.

### *Synthesis of 5-(4-acetoxybenzoylamino)isophthalohydrazide 28 and 5-(3-acetoxybenzoylamino)isophthalohydrazide 29 (General method)*

In a dry flask, 5-(4-acetoxybenzoylamino)isophthalic acid **8** (1 g, 2.9 mmol) dissolved in methanol (10 ml) was mixed with acetyl chloride (4 ml) and the mixture was refluxed for 3 h. the solvent was then removed under vacuum to furnish methyl 5-(4-acetoxybenzoylamino)isophthalate **26** as a white solid; m.p. 230°C. It was used as such in the next step. IR (υ cm^-1^): 4016, 3894, 3848, 3831, 3813, 3793, 3726, 3702, 3685, 3665, 3641, 3389, 3324, 3198, 2950, 2353, 1909, 1719, 1706, 1649, 1605, 1551, 1513, 1436, 1352, 1284, 1255, 1171, 1104, 1005, 907, 873, 845, 758, 719, 700, 672, 621, 586, 550, 515, 481, 456.

In a similar manner, methyl 5-(3-acetoxybenzoylamino)isophthalate **27** was prepared in quantitative yield from 5-(3-acetoxybenzoylamino)isophthalic acid **9**; m.p. 130°C. IR (υ cm^-1^): 4069, 3392, 3365, 3072, 2951, 2804, 2575, 1962, 1717, 1646, 1585, 1550, 1491, 1437, 1342, 1293, 1250, 1213, 1122, 1100, 992, 928, 903, 875, 836, 806, 793, 753,721, 679, 596, 568, 539, 513.

Methyl 5-(4-acetoxybenzoylamino)isophthalate **26** (1.1 g, 2.6 mmol) dissolved in 10 ml MeOH was treated with hydrazine hydrate (1.0 ml) and the mixture was refluxed for 2 h. Solvents and volatiles were removed under vacuum and the titled compound **28** was obtained as white solid; m.p. 250°C. IR (cm^-1^): 3303, 2828, 2697, 2624, 2517, 2354, 1654, 1591, 1554, 1512, 1428, 1357, 1309, 1253, 1183, 1118, 1007, 954, 891, 840, 737, 684, 592, 546, 484.

In a similar method, 5-(3-acetoxybenzoylamino)isophthalohydrazide **29** was prepared in quantitative yield from methyl 5-(3-acetoxybenzoylamino)isophthalate **27**; m.p. 150°C. IR (cm^-1^): 3454, 3371, 3254, 3091, 3012, 2959, 2851, 2358, 1935, 1719, 1647, 1602, 1442, 1365, 1253, 1104, 1004, 942, 893, 867, 788, 756, 723, 668, 577, 539.

#### Synthesis of poly 4-((3-(2-(3-acetylbenzoyl)hydrazinecarbonyl)-5-(2-methyl hydrazinecarbonyl)phenyl)carbamoyl)phenyl acetate 31 and poly 3-((3-(2-(3-acetylbenzoyl)hydrazine carbonyl)-5-(2-methylhydrazinecarbonyl)phenyl) carbamoyl)phenyl acetate 32

The bis-hydrazide **28** (1 g, 2.7 mmol) and isophthaloyl chloride **30**[19-22] (0.5 g, 2.4 mmol) in DMA (10 ml) was stirred at rt for 3 h and then poured on to cooled water. The polymer was collected filtration, washed with water, ethyl alcohol, water again and dried in a vacuum oven at 60°C for 24 h. The polyamide **31** was obtained in 94% yield (1.2 g); m.p. > 300°C, *η*_inh_ = 0.362 dL/g. Elemental analysis calculated for **31** (C_25_H_21_N_5_O_8_)_n_ : C, 57.80; H, 4.00; N, 13.48; Found: C,57.51 ; H, 4.28; N, 13.83. IR (υ cm^-1^): 3310, 2827, 2693, 2622, 2518, 2354, 2326, 1654, 1593, 1553, 1512, 1428, 1356, 1307, 1254, 1181, 1117, 1007, 955, 891, 841, 737, 687, 637, 594.

Polyamide **32** was prepared in a similar method starting by *5-(3-acetoxybenzoylamino) isophthalohydrazide***29** and *isophthaloyl chloride***30** in a 94% (1.2 g); m.p. > 300°C. Elemental analysis calculated for **32** (C_25_H_21_N_5_O_8_)_n_: C, 57.80; H, 4.00; N, 13.48; Found: C, 58.21; H, 3.69; N, 13.09. IR (υ cm^-1^): 3268, 2951, 2350, 2299, 1721, 1655, 1599, 1549, 1440, 1343, 1294, 1250, 1122, 998, 951, 901, 881, 838, 801, 754, 683, 600, 532.

#### Synthesis of 4-((3-(5-(3-acetylphenyl)-1,3,4-oxadiazol-2-yl)-5-(5-methyl-1,3,4-oxadiazol-2-yl)phenyl)carbamoyl)phenyl acetate 33 and 3-((3-(5-(3-acetyl phenyl)-1,3,4-oxadiazol-2-yl)-5-(5-methyl-1,3,4-oxadiazol-2-yl)phenyl)- carbamoyl)phenyl acetate 34

The polyhydrazide **31** (0.5 g, 0.96 mmol) was mixed with thionyl chloride (10 ml) and the mixture was refluxed for 6 h. The excess of thionyl chloride and volatiles were distilled off by and the resulted solid was treated with hexane and dried under vacuum to furnish the polymer **33**; m.p. > 300°C, *η*_inh_ = 0.341 dL/g. IR (υ cm^-1^): 4388, 4300, 4081, 3896, 3849, 3794, 3729, 3707, 3664, 3216, 3080, 2923, 2355, 2328, 1822, 1704, 1652, 1602, 1548, 1511, 1445, 1332, 1205, 959, 902, 845, 753, 668. Elemental analysis calculated for (C_26_H_17_N_5_O_7_)_n_: C, 61.05; H, 3.32; N, 13.69; Found: C, 59.83 ; H, 3.77; N, 13.42.

In a similar procedure, the polymer **34** was prepared from the polyhydrazide **32** and the following data were recorded: Yield: 87% (0.43 g); m.p. > 300°C, *η*_inh_ = 0.331 dL/g. IR (υ cm^-1^): 3436, 2925, 1717, 1657, 1594, 1554, 1445, 1342, 1295, 1256, 1127, 1001, 903, 839, 755, 680, 605. Elemental analysis calculated for **34** (C_26_H_17_N_5_O_7_)_n_: C, 61.05; H, 3.32; N, 13.69; Found: C,62.44; H, 3.74; N, 13.31.

### Polymer Particles Synthesis (General Method)

The diacid chloride **10** or **11** (0.5 mmol) and the appropriate diamine (0.5 mmol) were each dissolved in dioxane (50 mL). Distilled water (15 mL) was added to the solution of the diamine followed by the addition of the entire acid chloride solution at once. The resulted turbid solution was ultrasonicated at 42 KHz in a water bath for a period of 30 min. The polymer colloidal solution was extracted by centrifugal separation for 15 min. at 15,000 rpm and the resulted precipitate were carefully washed with methanol and water to purify the product of any unreacted monomer. The polymer samples were then dried at 60°C for 10 h then kept in a vacuum desiccator.

## Results and discussions

### Synthesis of polyamides containing pendent *m*- and *p*-acetoxybezamides groups

**Scheme 1:**
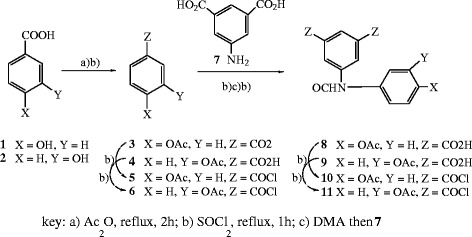
Preparation of 5-(4-acetoxybenzamido)isophthaloyl chloride 10 and 5-(3-acetoxybenzamido)isophthaloyl chloride 11.

**Figure 1:**
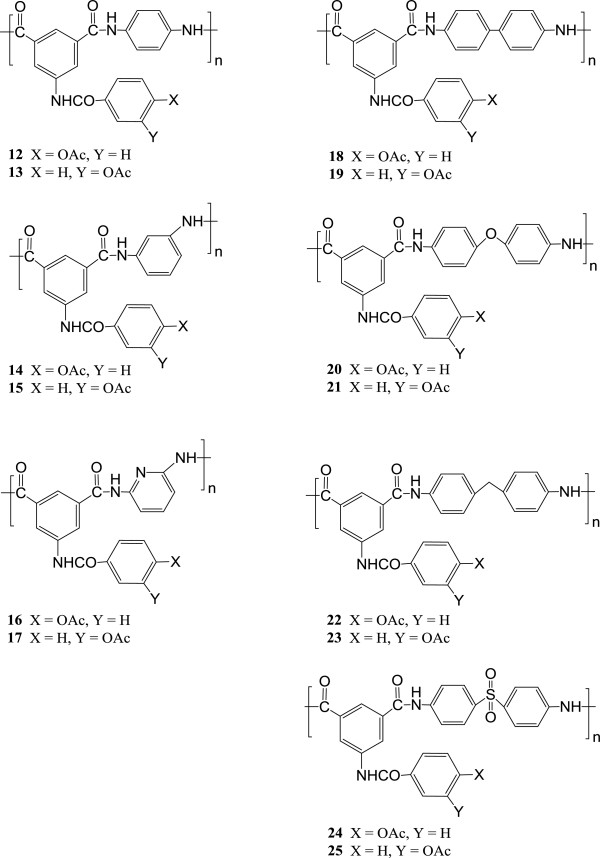
Chemical Structures of the prepared polyamides 12–25.

The production of new types of aromatic polyamides containing pendant structures comprised of *m*- and *p*-acetoxybenzamide groups, where the acetoxybenzamide groups act as signaling units due to their fluorescent and chromogenic characteristics, and studying of their properties is the major objective of our study. One of the most important tasks in this study is to analyze and predict their properties such as solubility, optical and fluorescence emission properties and thermal stability with respect to their chemical structure. 5-(4-Acetoxybenzamido)isophthaloyl chloride **10**[11] was synthesized by a sequence of reactions in which *p*-hydroxybenzoic acid **1** reacted with acetic anhydride to give *p*-acetoxybenzoic acid **3** that by further treatment with thionyl chloride gave the corresponding acid chloride; the latter reacted with 5-aminoisophthalic acid **7** to produce *p*-(acetoxybenzamido)isophthalic acid **8** which, by further treatment with thionyl chloride, gave *p*-(acetoxybenzamido)isophthaloyl chloride **10**, Scheme 1. Following similar sequence of reactions, 5-(3-acetoxybenzamido)isophthaloyl chloride **11** was prepared starting from *m*-hydroxybenzoic acid **2**. Polyamides **12–25** (Figure 1) were synthesized in moderate yields by direct solution polycondensation of an equimolar mixture of the acids chlorides **10–11** with *p*-phenylenediamine, *m*-phenylenediamine, 2,6-diaminopyridine, 4,4^′^-diaminodiphenylsulfone, 4,4^′^-diaminodiphenyl ether, 4,4^′^-diaminodiphenylmethane and benzidine in *N,N*-dimethylacetamide solution. The polymer structures were confirmed by elemental analysis, IR and UV–vis spectroscopy. Elemental analyses are in a good agreement with the proposed structures. A detailed description of the physical properties of the polymers **12–25** are given in the experimental section.

**Figure 2:**
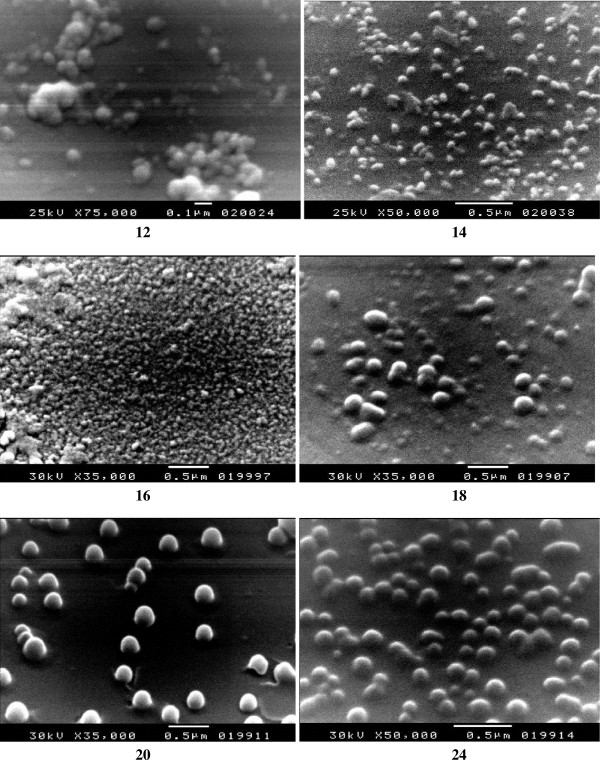
SEM images of the aramides nanoparticles 12, 14, 16, 18, 20 and 24.

Our attention focused next on the synthesis of new types of aramide nanoparticles containing bulky pendent groups. Polymeric aromatic nanoparticles can be prepared by either emulsion or interfacial polymerizations. Additionally, a popular method used for polymeric nanoparticles preparation is solvent displacement, also referred to as nanoprecipitation [24]. The basic principle of this technique is based on the interfacial deposition of a polymer after displacement of a semipolar solvent, miscible with water, from a lipophilic solution. Rapid diffusion of the solvent into the non-solvent phase results in a decrease of interfacial tension between the two phases, which increases the surface area and leads to the formation of small droplets of organic solvent. The key variables determining the success of the method and affecting the physicochemical properties of nanoparticles are those associated with the conditions of adding the organic phase to the aqueous phase, such as organic phase injection rate, aqueous phase agitation rate, the method of organic phase addition and the organic phase to aqueous phase ratio. Likewise, nanoparticle characteristics are influenced by the nature and concentration of their components [25,26]. The process of particle formation in the nanoprecipitation method comprises three stages: nucleation, growth and aggregation. The rate of each step determines the particle size and the driving force of these phenomena is the ratio of polymer concentration over the solubility of the polymer in the solvent mixture. The separation between the nucleation and the growth stages is the key factor for uniform particle formation [27]. As a representative example, the nanoparticles series **12, 14, 16, 18, 20** and **24** were prepared by ultrasonication of 0.5 mmol of *p*-phenylenediamine, *m*-phenylenediamine, 2,6-diaminopyridine, benzidine, diaminodiphenylether and diaminodiphenylsulfone, respectively, with 0.5 mmol of the acid chloride **10** in a total of 115 mL dioxane solution containing distilled water (15 mL) (*i.e.*, 50/15 mL dioxane-water *v/v* diamine solution and 50 mL dioxane acid chloride solution) followed by centrifugal separation at 15,000 rpm for 30 min. Mixing 1,4-dioxane with H_2_O was essential for many reasons, such as controlling the particle morphology, playing an important role in determining the polarity of the reaction solution and as a reaction accelerator [20-23]. As judged by SEM photographs, (Figure 2), the prepared polyamides were obtained as well-separated spherical nanoparticles while aggregated particles of the aramide containing pyridine **16** were obtained. The average diameters (standard deviation) were **12**; 67.5 nm (2.54), **14**; 55.86 (5.35), **16**; 39.65 nm (3.69), **18**; 135.14 nm (11.83), **20**; 130.50 nm (15.82) and **24**; 115.92 nm (10.37), respectively. The formation of such aggregate may be attributed to the molecular self assembly via H-bond directed organization of molecular precursors [28]. Amides embody self-complementary recognition groups defined by homomeric H-bond donor-acceptor pairs [29]. Noteworthy, addition of particular amount of water (15% v/v) to this reaction system is essential, not only for the formation of spherical particles, but also to diminish the aggregation of these particles. The tendency of spherical particles formation of such aramides may be correlated to the dispersion stability of particles in the reaction solution or the precipitation mechanism of the particles. The formation mechanism of polymers is related to the effect of water on the micelle structure. Micelles grow in the presence of water to spherical micelles [20-23].

**Scheme 2:**
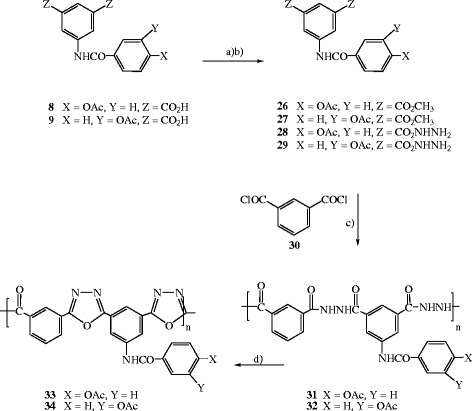
Synthetic pathways toward the hydrazide polymers 31, 32 and poly(1,3,4- oxadiazole-amide)s 33, 34.

5-(4-acetoxybenzoylamino)isophthalate **26** and methyl 5-(3-acetoxybenzoylamino)isophthalate **27** were prepared in quantitative yield from the corresponding acids **8** and **9**, respectively, Scheme 2. Boiling of the esters **26** and **27** with hydrazinehydrate furnished the expected 5-(4-acetoxybenzoylamino)isophthalohydrazide **28** and 5-(3-acetoxybenzoylamino)isophthalohydrazide **29**, respectively. The hydrazide polymers **31** and **32** were efficiently prepared in quantitative yields by solution polycondensation of the bis-hydrazides **28** and **29**, respectively, with the readily available isopthaloyl chloride **30**[19-22] in *N,N*-dimethylacetamide. The hydrazide polymers **31** and **32** were cyclodehydrated through heating with thionyl chloride for several hours to furnish the corresponding poly(1,3,4-oxadiazole-amide)s **33** and **34**, respectively. Probably due to the increased length of conjugated chain and/or the formation of charge-transfer complex between the oxadiazole ring and the aromatic unit, the hydrazide polymers **33** and **34** turned into darkened and deep brown after cyclodehydration. Conversion of the hydrazide group to the 1,3,4-oxadiazole ring could be monitored by FT-IR. Conversion of the acyl hydrazide group to the 1,3,4-oxadiazole unit can be confirmed by the disappearance of the N–H stretching absorption at 3252 cm^−1^ and the carbonyl peak at 1653 cm^−1^, together with the appearance of the oxadiazole characteristic bands at 1550–1570 cm^-1^, 1070 cm^−1^ and 980–990 cm^-1^, [30] with a broadening and minor shift of the bands in the polymer spectra. Other characteristic vibrations include the aromatic skeletal stretching at 1610 and 1480 cm^−1^. TGA, as discussed subsequently, was also used to investigate cyclization to the oxadiazole structure.

### Physical properties of the prepared polymers

#### Solubility

All of the polyamides **12–25** and polyhydrazides **31**–**32** were readily soluble in polar solvents such as NMP, DMAc, DMF and DMSO while insoluble in boiling alcoholic or halogenated solvents. The observed good solubility compared with that of related aromatic polyamides and aromatic polyoxadiazole-amides are due to the presence of voluminous acetoxybenzamide side groups which prevent the packing of the macromolecules through hydrogen bonds between amide groups in the chain. Due to the increased chain rigidity, the poly(amide-1,3,4-oxadiazole)s **33–34** showed a dramatically decreased solubility as compared to the corresponding hydrazide prepolymers. The oxadiazole based polymers were insoluble in all the organic solvents tested. This also indicates that these oxadiazole polymers exhibit good chemical stability.

#### Inherent viscosity

The inherent viscosity (*η*_inh_) of the polymers, as a suitable criterion for evaluation of molecular weight, was measured at a concentration of 0.5 g / dL in DMSO at 30°C. It was in the range of 0.359–0.765 dL/g that showed moderate molecular weights. From the data it is concluded that the prepared linear symmetric meta-polymers exhibited high *η*_inh_ values and thus high degree of polymerization.

#### FTIR spectroscopy

The FT-IR spectra of the prepared polymers exhibited characteristic absorbance at υ3300 and υ1650 cm^-1^ corresponding to the N–H and C = O stretching of amide group, respectively. Bands at υ3050 and υ1600 cm^-1^ were assigned to the aromatic H–C_str_ and C–C_str_, respectively. The absorption bands appearing at 1020 cm^-1^ and 960 cm^-1^ were characteristic of = C–O–C = stretching in 1,3,4-oxadiazole rings. A detailed description of the IR data of the polyamides **12–25** and **31–34** are given in the experimental section.

#### UV–vis and fluorescence emission studies

The optical properties of polymeric particles **12–25** and **31–34** were investigated by UV–vis spectroscopy in DMSO using a polymer concentration of ~ 2 mg / 10 mL. Comparison between the polyamide particles clearly revealed that the absorbance characteristics of the polymer are affected by the linear conjugated system. Spectral-luminescent characteristics of the prepared polymers **12–25** and **31–34** in DMSO are presented in Table 1. The studied polymers have wide absorption spectral bands with maxima situated between 276 and 325 nm, principally due to electronic transitions of electrons in long conjugated sequence of π-bonds (π → π* transitions) and electronic *n* → π* transitions of non-bonding lone-pair electrons on heteroatoms in π-bonds. The values of molar extinction coefficients are in the range from 2400 M^-1^ cm^-1^ to 25000 M^-1^ cm^-1^. For the polyamides containing *m*-acetoxybenzamide pendant groups the positions of absorption spectra maxima are shifted, as compared to those containing *p*-acetoxybenzamide pendant groups, to the short-wavelength region up to 10 nm. Interestingly, polymers **18** and **19** derived from benzidine exhibited additional long shoulders at λ 325 nm and λ 323 nm, respectively. The presence of additional bands in **18** and **19** absorption spectra could be due to the longer conjugation sequence of π-bonds compared to other polymers.

**Table 1:** Optical properities of polyamides 12-25, polyhydrazides 31, 32 and poly(1,3,4-oxadiazoles) 33, 34

**Polymer No**	^**a**^λ_**abs **_**(nm)**	**ε**^**b **^**(M**^**-1**^ **cm**^**-1**^**)**	^**c**^λ_**em **_**(nm) (**^**d**^λ_**ex **_**at 300 nm)**
**12**	292	2400	345, 647
**13**	288	4300	361, 696
**14**	286	20000	343, 642
**15**	278	7100	341, 650
**16**	284	12000	345, 650
**17**	276	4600	338, 646
**18**	297	17200	364, 407
	325	18100	420, 744
**19**	285	6900	362, 644
	323	9000	
**20**	295	23500	347, 642
**21**	291	8500	340, 652
**23**	282	8700	341, 646
**24**	309	25000	346, 658
**25**	286	5200	336, 376
	305	5700	400, 684
**31**	280	7700	357, 502
**32**	274	8400	363 (w), 427
**33**	283	10000	360 (w), 431
**34**	277	6800	363 (w), 428, 529 (w), 637(sh)

^a^λ_abs_: maximum wavelengths of absorption.

^b^ε: the molar absorption coefficient calculated from *Beer-Lambert equation*

(A = ε x *c* x *l*); where A is absorbance, *c* is concentration, *l* = 1 cm).

^c^λ_em_: maximum wavelengths of emission.

^d^λ_ex_: maximum wavelengths of excitation.

**Figure 3:**
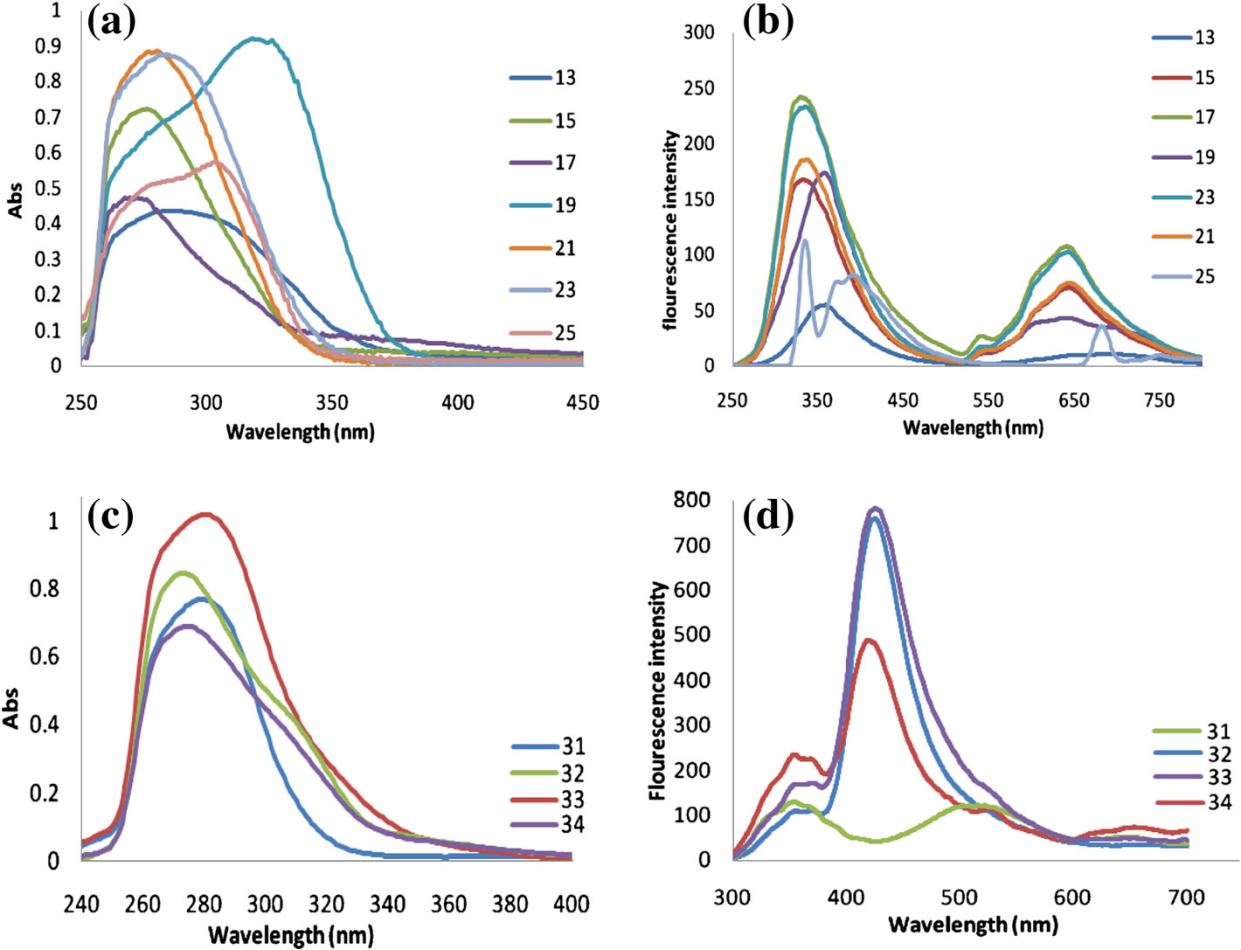
UV–vis absorption images and fluorescent spectra of the polyamides 13, 15, 17, 19, 21, 23, 25.

The PL spectra were recorded at 300 nm excitation wavelength using a polymer concentration of (10^-5^) (2 mg / 10 ml (DMSO) diluted up to 100 ml). The excitation maxima positions of the studied polyamides containing *m*-acetoxybenzamide groups are shifted to the lower-wavelength spectral region relatively to those containing *p*-acetoxybenzamide pendant groups. In general, most polyamides exhibited two excitation maxima; long-wavelength bands situated in the range 696–642 nm and lower-wavelength bands in the range 362–338 nm. In the case of polyamides **18** and **25** four excitation maxima were observed. Polymer 18 exhibited the main bands with maximum at 744 nm, 420 nm, 407 nm and 336 nm while polyamide **25** showed bands at 686 nm, 400 nm, 376 nm and 336 nm. Figure 3 shows the UV–vis absorption images and fluorescent spectra of selected polyamide series, polyhydrazides **31, 32** and poly(1,3,4-oxadiazole-amide)s **33, 34**.

Polyhydrazides **31** and **32** exhibited major absorption bands at 280 nm and 274 nm, respectively, mainly due to π-bonds (π → π* transitions) of the aromatic units. In addition, **31** and **32** exhibited two major excitation maxima (recorded at 300 nm) at 502 nm and 427 nm and low intensity shoulders at 357 nm and 363 nm, respectively. Poly(1,3,4-oxadiazole-amide)s **33** and **34** showed two strong UV absorption peaks at 283 nm and 277 nm, respectively. The PL spectra of **33** and **34** showed two strong excitation peaks at 431 nm and 428 nm, in addition, to two little excitation bands at 360 nm and 363 nm, respectively. A weak excitation band at 529 nm and a little shoulder at 637 nm were also observed for the oxadiazole **34**.

From the electronic and spectral points of view, the oxadiazole ring is similar to a *para-*phenylene structure and has a strong electron-withdrawing character. Due to the similarity with *para*-phenylene unit, known as highly thermoresistent, the 1,3,4-oxadiazole ring is called *pseudophenylene*[31]. However, the introduction of 1,3,4-oxadiazole rings in the macromolecular chains motivated by many other features such as: *(a) *1,3,4-oxadiazole ring is free of hydrogen and, therefore, poly(1,3,4-oxadiazole)s maintain their properties during heating, in air, better than other polymers; *(b)* conjugation between 1,3,4-oxadiazole and other aromatic rings in polymers is similar to that of polyphenylenes; *(c) *1,3,4-oxadiazole ring is free of tension; *(d)* 1,3,4-oxadiazole cycle doesn't have any possibilities of rearrangement; *(e)* 1,3,4-oxadiazole ring has a structural symmetry and it is thermally unreactive. From the data given in Table 1, we noticed that the absorption maxima of polymers **12, 13** (*p*-phenylene-containing polymers) are comparable with that of oxadiazole-containing polymers **33**, **34**. Polymers-containing oxadiazoles units **33**, **34** present strong excitation peaks around 431 and 428 nm, in addition, two lower excitation bands at 360, 363 nm, respectively, while the major excitation bands in case of **12, 13** were found at lower wavelengths at 345, 361 nm, respectively, in addition, two low intensity excitation peaks at 647, 696 nm, respectively. Solely, polymer **34** exhibit additional peaks at higher wavelengths at 529 nm (weak band) and 637 nm (weak shoulder) may be due to other chromophores present in the macromolecular chain.

Structure-PL property relationship may achieved further trends and conclusions:

1. Polyamides **14** and **16**, containing *p*-acetoxybenzamide pendant groups, derived from *m*-phenylenediamine and 2,6-diaminopyridine, respectively, exhibited almost similar strong emission peaks at about 343 nm and 345 nm. However, their analogues **15, 17** containing *m*-acetoxybenzamide groups exhibited slightly blue-shifted peaks (shift to a lower wavelength up to 7 nm) at 341 nm and 338 nm, respectively. Thus, no significant changes in the absorption spectra were noticed upon replacing the phenylene units by pyridine units in the macromolecular chain.

2. The blue emissions at 420 nm and 405 nm for the polyamide **18** derived from benzidine, attributed to the highly conjugation system, also blue-shifted (shifted to a lower wavelength) upon introducing flexible linkages such as ether bond in **20** (314 nm), methylene group in **23** (336 nm) or sulfone group in **24** (344 nm) in the polymeric chains.

3. Blue emission observed for benzidine-containing polyamide **18** containing *p*-acetoxy substituent (407 nm) was dramatically blue-shifted (shifted to a lower wavelength) (362 nm) for its analogue **19** containing *m*-acetoxy substituent. This result clearly demonstrated that methoxy group behaves differently depending upon how it is structurally oriented. Both the resonance (+ *R*) and inductive (−*I*) effects of the *p*-acetoxy substituents account for the observed high wavelength emission in **18** while only inductive effect (−*I*) of the *m*-acetoxy substituents account for relatively lower wavelength emission peak in **19**.

#### Thermal analysis

**Figure 4:**
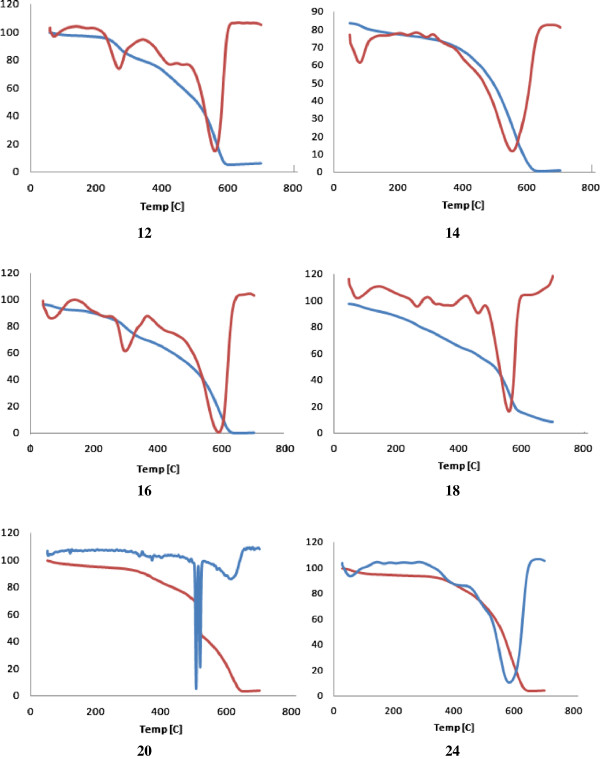
TG/DTG curves of the polymeric nanoparticles 12, 14, 16, 18, 20, 24.

**Figure 5:**
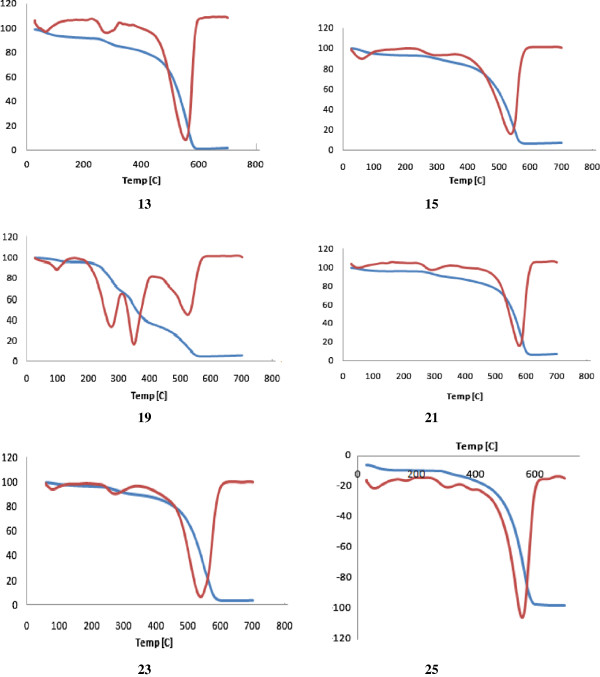
TG/DTG curves of the polymeric nanoparticles 13, 15, 19, 21, 23, 25.

The thermal properties of the prepared polymers were evaluated by differential thermo gravimetric (DTG) and differential thermal analysis (DTA) techniques. Thermal stability of the polymers was studied in the range 25°C – 700°C (char yield) and the results are complied in Tables 2 and 3. Thermal results revealed that the polyamides **12, 14, 16, 18, 20** and **24** derived from the diacid chloride **10** demonstrated higher thermal stability compared to their partners **13, 15, 17, 19, 21** and **25**, respectively, derived from the diacid chloride **11**. All polymers exhibited an endothermic decomposition peaks in the range 80°C – 150°C correspond to a dehydration process while the major decomposition peaks appeared around 524°C – 700°C which may be attributed to the cleavage of the amide bonds. Structure-thermal property correlation based on changing the diamine monomer, as a single structural modification, demonstrated an interesting connection between a single change and thermal properties. TG/DTG curves of the polymeric nanoparticles **12–24** are shown in Figures 4 and 5. Polyamides **14**, derived from *m*-phenylenediamine, exhibited three endothermic decomposition peaks at 150°C, 286°C and 552°C leaving 17% of the polymer as remaining mass residue; while polyamide **16**, derived from 2,6-diaminopyridine showed four exothermic decompositions peaks at 120°C, 200°C, 300°C and 590°C, respectively, leaving 3.6% of the polymer as residue. This result clearly demonstrated the high thermal stability of **14** relative to its analogues **16**. Polyamides **18, 20, 24** exhibited major degradation processes at 560°C, 614°C and 584°C leaving 11.9%, 3.9% and 6.0% of the polymers, respectively, as char yields. Similarly, polyamides **19, 21, 25** exhibited major degradation processes at 578°C, 605°C and 556°C leaving 6.9%, 8.1% and 8.3% char yields.

**Table 2:** Thermoanalytical data of the prepared polymers 12–25

**No**	**DTG (°C)**	**Wt.loss (%)**	**Residue (%)**	**LOI**^**a**^	**No**	**DTG (°C)**	**Wt.loss (%)**	**Residue (%)**	**LOI**^**a**^
**12**	268	16.7	6.90	19.7	**18**	265	18.3	11.93	21.7
	425	22.0				350	15.5		
	561	54.4				461	8.8		
						560	45.4		
**13**	279	14.2	3.70	18.4	**19**	291	10.1	6.98	19.7
	553	82.1				578	82.8		
**14**	286	9.3	17.17	23.8	**20**	332	9.0	3.97	18.5
	552	73.5				370	18.4		
						506	15.7		
						614	38.9		
**15**	294	12.5	6.30	19.5	**21**	367	13.2	8.10	20.2
	540	81.2				605	78.6		
**16**	206	10.4	3.62	18.4	**23**	274	9.7	3.83	18.5
	296	17.6				539	86.4		
	590	68.3							
**17**	275	29.6	5.32	19.1	**24**	389	15.9	6.04	19.4
	348	32.1				584	77.9		
	524	32.9							

^a^limiting oxygen index.

**Table 3:** Thermoanalytical data of the polyhydrazides 31, 32 and poly (1,3,4-oxadiazole-amide)s 33, 34

**No**	**DTG (°C)**	**Wt.loss (%)**	**Residue (%)**	**LOI**^**a**^
**31**	308	19.0	7.61	20.0
	549	73.3		
**32**	334	37.5	1.68	17.6
	405	20.0		
	574	40.7		
**33**	232	18.5	6.23	19.4
	443	24.8		
	570	50.3		
**34**	258	34.8	2.64	18.0
	318	31.3		
	551	31.3		

^a^limiting oxygen index.

**Figure 6:**
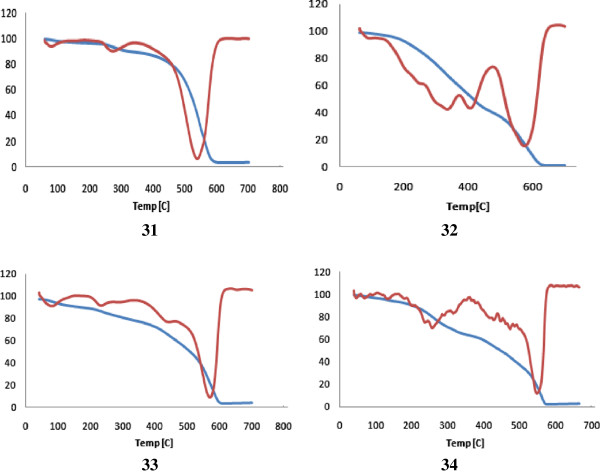
TG/DTG curves of the polyhydrazides 31, 32 and poly(1,3,4-oxadiazole-amide)s 33, 34.

Polyhydrazide **31** exhibited three degradation processes at 130°C, 308°C and 549°C, respectively, leaving 7.6% char yields while degradation processes of the polymer **32** occurred at 130°C, 334°C, 574°C, respectively, and only 1.6% of the polymer was the remaining residue. Interestingly, polyhydrazides **31** and **32** exhibited second stages weight losses 15.35% at 308°C and 35.36% at 334°C, respectively, attributed to the loss of the terminal OAc and dehydrative cyclization (15.70%) in case of **31** while debloking of the pendant group (35.38%) in case of polymer **32**. These results clearly proved that the polyhydrazide **31** derived from the diacid chloride **10** exhibited higher thermal stability compared to its partner **32** derived from the diacid chloride **11**. TG/DTG curves of the polymeric nanoparticles **31–34** are shown in Figure 6.

Char yield can be used as criteria for evaluating limiting oxygen index (LOI) of the polymers in accordance with Van Krevelen and Hoftyzer equation [32]. LOI = 17 + 0.4CR where CR = char yield. The LOI values of all polymers calculated based on their char yield at 700°C were less than 28. On the basis of LOI values, such macromolecules cannot be classified as self-extinguishing polymers.

The thermodynamic parameters of decomposition processes of polymers, namely, activation energy Δ*E* enthalpy (Δ*H*), entropy (Δ*S*) were evaluated by employing the Horowitz-Metzger equation [33], Tables 4 and 5. The order of chemical reactions (n) was calculated via the peak symmetry method by Kissinger [27]. The asymmetry of the peak, *S*, is calculated as follows:

(1) $$S=0.63n^{2}$$

(2) $$n=1.26{\left(a/b\right)}^{1/2}$$

**Table 4:** Kinetic parameters of the polyamides 12–25

**No**	**Slope DTA**	**ΔE**^**a**^	**a**	**b**	**S**^**b**^	**N**^**c**^	**α**_**m**_^**d**^	**T**_**m **_**(K)**	**Z**^**e**^	**ΔS**^**f**^	**ΔH**^**g**^	^**h**^**R**^**2**^
**12**	−23.5	195.5	1.3	0.7	5.3	1.7	0.5	537.7	3.9	−0.23	−128.1	0.96
	−11.1	93.0	2.9	1.5	5.2	1.7	0.5	833.5	1.1	−0.25	−210.3	0.97
**13**	−12.7	105.6	3.6	1.2	8.5	2.1	0.4	823.4	0.7	−0.25	−210.2	0.95
**14**	−10.5	87.7	1	0.7	4.0	1.5	0.5	815.7	1.0	−0.25	−205.9	0.99
	−9.4	78.5	6	3	5.7	1.7	0.5	821.2	0.9	−0.25	−208.2	0.96
**15**	−17.4	145.0	4	1.9	6.0	1.8	0.5	810.1	1.8	−0.24	−201.0	0.99
**16**	−29.4	244.7	0.6	0.5	3.4	1.3	0.5	648.4	4.0	−0.23	−155.4	0.96
	−17.0	141.9	4.8	1.6	8.5	2.1	0.4	867.7	1.6	−0.24	−216.5	0.98
**17**	−10.2	85.1	0.9	0.6	4.2	1.5	0.5	405.1	1.3	−0.24	−99.2	0.98
	−23.6	196.9	0.6	0.7	2.4	1.1	0.6	554.2	2.3	−0.24	−134.7	0.97
	−11.8	98.8	0.4	1	1.1	0.7	0.6	629.3	0.9	−0.25	−158.1	0.98
	−53.9	448.6	3	1.5	5.7	1.7	0.5	801.9	3.6	−0.24	−194.3	0.99
**18**	−20.7	172.3	0.4	1.2	0.9	0.7	0.6	365.8	5.5	−0.23	−85.0	0.86
	−36.3	302.4	1.3	0.7	5.3	1.7	0.5	736.5	4.4	−0.24	−176.8	0.97
	−7.3	60.8	2.3	1.1	4.1	1.8	0.5	836.3	0.7	−0.25	−214.1	0.82
**19**	−23.6	196.9	3.4	1.5	6.4	1.8	0.5	852.2	2.3	−0.24	−209.9	0.99
**20**	−40.5	337.8	0.9	0.5	5.1	1.6	0.5	790.3	4.5	−0.24	−189.9	0.95
	−25.0	207.8	2.8	1.6	5.0	1.6	0.5	887.6	2.4	−0.24	−218.9	0.86
**21**	−21.9	182.6	0.5	0.6	2.2	1.1	0.6	679.7	2.8	−0.24	−165.2	0.99
	−11.7	97.5	4.5	1.7	7.5	2.0	0.4	880.1	1.1	−0.25	−222.6	0.90
**23**	−14.1	117.4	4.2	2.4	5	1.6	0.5	815.5	0.8	−0.25	−207.3	0.99
	−4.6	38.8	4	1.6	7.1	1.9	0.5	834.3	0.2	−0.26	−220.2	0.91
**24**	−25.6	213.1	0.8	0.4	6.0	1.8	0.5	706.7	3.1	−0.24	−171.3	0.99
	−24.8	206.8	3.4	2.5	3.8	1.4	0.5	852.4	2.5	−0.24	−209.6	0.92
**25**	−27.4	227.8	2.8	1.7	4.7	1.6	0.5	831.7	2.8	−0.24	−203.5	0.84

^a^DE:activation energy, ^b^S: asymmetry of the peak, ^c^n: order of chemical reaction, ^d^α_m_: decomposed substance fraction at the moment of maximum development of reaction with (T = T_m_),^e^Z: collision factor (s^-1^), ^f^ΔS: entropy in KJ/mol, ^g^ΔH: enthalpy in KJ/mol, ^h^R^2^: coefficient of determination.

**Table 5:** Kinetic parameters of the polyhydrazides 31, 32 and poly (1,3,4-oxadiazole-amide)s 33, 34

**No**	**Slope DTA**	**ΔE**^**a**^	**a**	**b**	**S**^**b**^	**N**^**c**^	**α**_**m**_^**d**^	**T**_**m **_**(K)**	**Z**^**e**^	**ΔS**^**f**^	**ΔH**^**g**^	^**h**^**R**^**2**^
**31**	−25.9	215.5	0.5	0.4	3.5	1.4	0.56	557.2	2.5	−0.24	−135.0	0.99
	−16.6	138.4	0.9	0.4	6.4	1.8	0.51	696.0	1.2	−0.25	−174.1	0.99
	−13.7	114.2	3.4	1.3	7.4	2.0	0.49	825.2	0.8	−0.25	−210.2	0.99
**32**	−19.7	164.3	4.3	2.3	5.3	1.7	0.52	853.9	1.1	−0.25	−215.3	0.99
**33**	−2.4	19.9	0.5	1.6	0.8	0.7	0.69	357.1	0.34	−0.25	−91.1	0.90
	−12.9	107.8	4.6	1.4	9.3	2.2	0.47	849.9	0.77	−0.25	−217.3	0.88
**34**	−19.5	162.8	3.2	1.1	8.3	2.1	0.48	823.0	1.2	−0.25	−207.1	0.94

^a^ΔE:activation energy, ^b^S: asymmetry of the peak, ^c^n: order of chemical reaction, ^d^α_m_: decomposed substance fraction at the moment of maximum development of reaction with (T = T_m_), ^e^Z: collision factor (s^-1^), ^f^ΔS: entropy in KJ/mol, ^g^ΔH: enthalpy in KJ/mol, ^h^R^2^: coefficient of determination.

The value of the decomposed substance fraction, α_m_, at the moment of maximum development of reaction (with T = Tm) being determined from the relation (3) [34]:

(3) $$\left(1-\alpha_{m}\right)=n^{1/1-n}$$

The values of collision factor, Z, can be obtained in case of Horowitz Metzger by making the use of the relation (4) [35]:

(4) $$Z=\frac{E}{RT_{m}}\phi \;\exp\left(\frac{E}{\mathrm{RT}\underset{m}{2}}\right)=\;\frac{KT_{m}}{h}\;\exp\;\left(\frac{\Delta S^{\#}}{R}\right)$$

where Δ*S*^*#*^ is the entropies of activation, R represents molar gas constant, ϕ rate of heating (K s^-1^), K the Boltzmann constant, and *h* the Planck’s constant [36]. The change in enthalpy (ΔH) for any phase transformation [37] taking place at any peak temperature, Tm, can be given by the following equation: ΔS = ΔH/T_m_. Based on least square calculations, the Ln ΔT versus 1000 / T plots for all complexes, for each DTA curve, gave straight lines from which the activation energies were calculated according to the reported methods [38]. The slope is of Arrhenius type and equals to -E/R.

The kinetic data obtained from the nonisothermal decomposition of the prepared polyamides **12–25** are given in Table 2. Some trends and conclusions may be achieved as follows:

1- The calculated values of the collision number, *Z*, showed a direct relation to *E*_a_. The maximum and minimum *Z* values are 5.51 and 0.78, respectively, to suggest different mechanisms with variable speeds. The values of the decomposed substance fraction, α_m_, at the maximum development of the reaction are of nearly the same magnitude and lie within the range 0.48–0.68. The maximum and the minimum *T*_m_ values are 887 K and 823 K, respectively.

2- The change of entropy values, ΔS, for all complexes are nearly of the same magnitude and lie within the range 0.23 to −0.26 kJ K^-1^ mol^-1^, all are with -ve signs. Therefore, the transition states are more ordered, i.e., in a less random molecular configuration than the reacting complexes. The fractions appearded in the calculated order of the thermal reactions, n, Table 4, confirmed that the reactions proceeded in complicated mechanisms.

3- Activation energies values (Δ*E*) of polyamides **12, 14, 16, 18, 20** and **24** derived from the diacid chloride **10** demonstrated lower Δ*E* values compared to their partners **13, 15, 17, 19, 21** and **25**, respectively, derived from the diacid chloride **11**. Noteworthy mentioning that sulfone-containing polymer **24, 25** exhibited higher ΔE than their ether-containing polymers analogs **20, 21**, respectively, and the first and second decomposition steps in some polymers and have nearly equal ΔE values, indicating similar degradation mechanism in both compounds. From the ΔE values, one can concluded that the water molecules are easily eliminated from all ligands and the energies of activation for the second stages of decomposition are higher than that of the first stage.

4- The enthalpy (Δ*H*) of polyamides **12, 14, 16, 18, 20** and **24** derived from the diacid chloride **10** demonstrated higher values compared to their partners **13, 15, 17, 19, 21** and **25**, respectively, derived from the diacid chloride **11** and the negative values of Δ*H* means that the decomposition processes are exothermic.

5- The collision number *Z* values of the polyhydrazides **31, 32** and their corresponding poly (1,3,4-oxadiazole) **33** and **34**, Table 3, are 0.84 and 1.19, 0.77 and 1.22, respectively, suggesting similar mechanisms. The values of the decomposed substance fraction, α_m_, at the maximum development of the reaction are of nearly the same magnitude and lie within the range 0.48–0.52. The maximum and the minimum *T*_m_ values are 849 K and 823 K, respectively. Activation energies values (Δ*E*) of polyamides **32** and **34** demonstrated higher Δ*E* values compared to their partners **31** and **33**, respectively. The enthalpy (Δ*H*) of polyamides 31–34 were −210 kjmol^-1^, -215 kjmol^-1^, 217 kjmol^-1^ and 207 kj mol^-1^, respectively, and the decomposition processes are exothermic.

## Conclusions

A series of aromatic polyamides nanoparticles with pendant structures comprised of *m*- and *p*-acetoxybenzamide groups were synthesized by solution polycondensation of 5-(4-acetoxybenzamido)isophthaloyl chloride or 5-(3-acetoxybenzamido)isophthaloyl chloride with commercially available aromatic diamines. In addition, two new poly(amide-1,3,4-oxadiazole)s were prepared via dehydrative cyclization of the copolyhydrazides derived from polycondensation of the readily accessible 5-(4-acetoxybenzoylamino) isophthalohydrazide or 5-(3-acetoxybenzoylamino) isophthalohydrazide, respectively, with isopthaloyl chloride. The thermal behavior of all polymers exhibited two major thermal decompositions at about 300°C, due to the breakage of the acetoxy group in the lateral chain, and at around 524°C – 700°C, which may be attributed to the cleavage of the main amide bonds. Structure- photoluminescence correlation based on changing the diamine monomer, as a single structural modification, demonstrated an interesting connection between a single change and optical and fluorescence emission properties. The blue emissions at 420 nm and 405 nm for the polyamide derived from benzidine, attributed to the highly conjugation system, was blue-shifted (shifted to a lower frequency) compared with that of polyamides containing flexible linkages. The prepared polymers could be dissolved in polar aprotic solvents at room temperature or upon heating. Further investigations to obtain films with reasonably good mechanical properties for different applications are in progress.

## Competing interests

The authors declare that they have no competing interests.

## Authors’ contributions

HHAMH and EMEM initiating the research point and monitored the experimental work, acquisition of data, analysis and interpretation of data and wrote draft the manuscript and gave final approval of the version to be published. AFEH carried out the thermal studies, acquisition of data, analysis and interpretation of data and helped to draft the manuscript. YAEK carried out the synthesis of monomers and polymers. All authors read and approved the final manuscript.

## Authors’ information

Part of M.Sc thesis of Y. M. A. Elkony.
